# Social media and anti-immigrant prejudice: a multi-method analysis of the role of social media use, threat perceptions, and cognitive ability

**DOI:** 10.3389/fpsyg.2024.1280366

**Published:** 2024-03-13

**Authors:** Saifuddin Ahmed, Kokil Jaidka, Vivian Hsueh Hua Chen, Mengxuan Cai, Anfan Chen, Claire Stravato Emes, Valerie Yu, Arul Chib

**Affiliations:** ^1^Wee Kim Wee School of Communication and Information, Nanyang Technological University, Singapore, Singapore; ^2^Department of Communications and New Media, National University of Singapore, Singapore, Singapore; ^3^Department of Media and Communication, Erasmus University Rotterdam, Rotterdam, Netherlands; ^4^Department of Communication Studies, Hong Kong Baptist University, Kowloon, Hong Kong SAR, China; ^5^International Institute of Social Studies, Erasmus University Rotterdam, Rotterdam, Netherlands

**Keywords:** social media, realistic threat, symbolic threat, cognitive ability, emotion, prejudice, immigrant

## Abstract

**Introduction:**

The discourse on immigration and immigrants is central to contemporary political and public discussions. Analyzing online conversations about immigrants provides valuable insights into public opinion, complemented by data from questionnaires on how attitudes are formed.

**Methods:**

The research includes two studies examining the expressive and informational use of social media. Study 1 conducted a computational text analysis of comments on Singaporean Facebook pages and forums, focusing on how social media is used to discuss immigrants. Study 2 utilized survey data to examine the use of social media at the individual level, testing the relationships between cognitive ability, perceptions of threat, negative emotions towards immigrants, and social media usage within the Integrated Threat Theory framework.

**Results:**

Study 1 found that discussions about immigrants on social media often involved negative emotions and concerns about economic impact, such as competition for jobs and crime. Complementing these findings about perceived economic threats, Study 2 showed that individuals with higher social media usage and greater perceptions of threat were more likely to have negative emotions towards immigrants. These relationships were mediated by perceptions of threat and were stronger in individuals with lower cognitive abilities.

**Discussion:**

The findings from both studies demonstrate the role of social media in shaping public attitudes towards immigrants, highlighting how perceived threats influence these attitudes. This research suggests the importance of considering how digital platforms contribute to public opinion on immigration, with implications for understanding the dynamics of attitude formation in the digital age.

## 1 Introduction

In recent years, the scale of international migration has increased due to major displacement events worldwide. Immigration is arduous for immigrants, but it is also regarded as a threat by indigenous societies (Pew Research Centre, 2019; Jedinger and Eisentraut, 2020; Lee et al., 2020) whose fear adverse personal consequences because of incoming immigrants (D’Errico and Paciello, 2018). Immigration policies are often sources of contention in countries with large migrant populations, such as the United States, Canada, Germany, the United Kingdom, Australia, and Singapore (United Nations, 2020, pp. 75). In this context, the Integrated Threat Theory (ITT) (Stephan and Stephan, 1996; Stephan and Stephan, 2000) offers a way to understand how the threat perceptions of the citizens in these countries may predict their prejudice towards immigrants (Stephan et al., 1999b). For instance, locals may perceive “symbolic” threats to their culture because of the different immigrant life habits and ways (see Stephan et al., 1999b). They may also perceive a “realistic” threat to economic stability because of the possible drop in job opportunities (Stephan et al., 1999b). Symbolic and realistic threats can manifest in social media, influencing individuals’ attitudes toward immigrants (Croucher et al., 2020; Mahmood et al., 2021). However, eliciting public opinion on immigrants and immigration is fraught with challenges. For instance, a social desirability bias and interviewer effects could lead to participants sharing mostly positive views toward immigrants. Even if this were not the case, while national surveys can offer more insights into public attitudes, the insights are limited to the items in the survey questionnaire, often without going into the reasons underlying those attitudes or the context in which the answer may have been given.

Mixed-method approaches to studying public opinion combine survey-based approaches with observational studies of social media posts (Maragoudakis et al., 2011; Chen and Tomblin, 2021). In doing so, they can triangulate the findings from analyses of both, how social media users use social media to inform themselves, or how they actively post on social media to express themselves. Such research designs can alleviate some of the concerns about studies reporting only survey methods, such as social desirability biases, and those reporting only social media-based analyses, such as selective participation or non-representativeness. These studies also allow researchers to consider both the *expressive* and the *informational* paradigms of social media use as factors associated with public opinion and social attitude formation. The expressive use of social media focuses on users’ self-expression behaviors, such as communicating their identity, opinions, and emotions in online conversations, replies, and reactions to posts (Macafee and De Simone, 2012). On the other hand, the information use of social media involves individuals’ consumption behaviors, such as following news outlets and subscribing to educational channels to seek information. Understanding the informational use of social media is crucial, because on the one hand, in a low-press freedom environment, the chilling effects of governmental surveillance may imply that Singaporeans are reluctant to share their opinion on political hotbed issues, such as the immigrant workforce. On the other hand, the proliferation of antisocial elements online under the guidance of anonymity may imply that the hate speech encountered online is out of proportion with the actual distribution of attitudes amongst the population. Therefore, informational use would be more typical of an average social media user, who consumes ‘filtered’ content, often endorsed by and sourced through their trusted peers.

We situate the current study within Singapore, a society where the expressive use of social media can offer critical insight into the integration of immigrants given the high-censorship, low-press freedom environment. Freedom scores, conducted by the Freedom House, evaluate the status of political rights and civil liberties in countries worldwide. Singapore ranked 47th out of 100 countries in the 2023 freedom scores (Freedom House, 2023). In Singapore, immigrants constitute 37.8% of the population (Statista, 2019). Previous work has reported that foreign workers in Singapore face the brunt of resentment related to their perceived dominance in the job market (McNulty, 2016) and their effect on overcrowded public resources (Dirksmeier, 2020). Some scholars have suggested that Singaporeans’ everyday lives are heightened with anti-immigrant emotions, xenophobia, and racial tension (Gomes, 2014; Velayutham, 2017; Yang, 2018), and they often turn to social media to express their emotional grievances toward immigrant groups (Velayutham, 2017). In 2019, the government acknowledged the danger of inflammatory social media discourse to racial harmony (Ministry of Home Affairs, 2019). Despite the rising concern, there is a lack of quantitative, empirical evidence to understand attitudes toward immigrants and assess the merit of the above arguments. In the following paragraphs, we motivate our key research questions and hypotheses guiding the study of the collected data before discussing the two research designs employed to analyze attitudes on social media.

## 2 Background

Large-scale social media data collection offers an alternative to understanding public opinion via the expressive use of social media, which provides a platform for individuals’ self-expression. When an individual expresses themself on social media, the act can clarify and reinforce their personal opinion (Cho et al., 2018). This is especially critical when studying interpersonal attitudes such as stereotypes and prejudice, as online anonymity would allow individuals to feel less inhibited and more comfortable sharing their innermost thoughts and desires (Theocharis et al., 2016; Oz et al., 2018). Social media platforms are also unmoderated or loosely moderated, implying that “uninhibited” posts invoking racist stereotypes and hate speech can proliferate on them (Weimann, 2010; D’Errico and Paciello, 2018). Moreover, social media platforms also allow hate speech to be transmitted to a nationwide audience (Hawdon et al., 2017). As a result of this hate speech on social media, the polarization of public opinion would increase, and individuals’ perceived social cohesion would also be negatively affected (Schäfer et al., 2021). In addition, previous research has found that exposure to hate materials online is associated with the occurrence of violence (Kiilakoski and Oksanen, 2011). Although social media discourse constitutes a non-representative sample, they are insightful in identifying majoritarian and emerging views put forth by “the politically active internet users (who) act like opinion-makers” and “who can influence (or to ‘anticipate’) the preference of a wider audience” (Ceron et al., 2014, pp. 345). In contrast, most traditional surveys consider that each individual is equivalent to a single data point, absent their sociocultural influence.

Conceptually, previous research in mining social media data for public opinion has exemplified two primary goals for mining social media users’ emotions and concerns. The first objective is to characterize the affective and cognitive factors underpinning political attitudes. For instance, Grover et al. (2019) found that pro-immigration tweets in the US were closely related to moral foundations of harm, fairness, and loyalty language elements, while anti-immigration tweets were associated with moral foundations of authority and negative emotions. Bartlett and Norrie (2015) surveyed tweets regarding the 2013–2014 Immigration Bill in the United Kingdom and found that half were associated with negative emotions. Furthermore, in a content analysis of Facebook posts by political parties across six European countries, Heidenreich et al. (2020) reported greater negative emotion in discussions about immigrants by political parties on the extreme left and right. In the absence of relevant research on users’ attitudes towards immigrants in Singapore, we therefore pose our first research question:

*RQ1:* What is the primary emotion in discussions about immigrants on social media?

The second goal of the computational text analysis of social media posts involves identifying the major priorities and concerns in civic discussions. Topic, opinion, and emotion detection from social media content allow scholars and political analysts to identify emerging social trends and public responses to specific events or policies (Sobkowicz et al., 2012). Looking at the emerging discussion topics over time can inspire policies and guide interventions. Previous studies have applied topic modeling to identify individuals’ issues online (Maragoudakis et al., 2011). Similar approaches offer insights about public concerns in a presidential election (Karami et al., 2018) and the Affordable Care Act (Hopkins and Parish, 2019) – but once again, there is a lack of research focus on the discussions around immigrants, thereby motivating our second research question:

*RQ2:* What are the topical themes in discussing immigrants on social media?

Motivated by prior multi-method analyses of social media users, the second part of our research project delves into the *informational* use of social media, different from the *expressive* use as it would encompass social media users who may not post, yet are avid consumers of social media content. Informational use of social media, different from expressive use, focuses more on seeking factual information, news, and educational content. Compared to expressive use, informational use emphasizes acquiring and sharing knowledge rather than personal feelings (Macafee and De Simone, 2012).

The emotional tone of social media content is pivotal in shaping attitudes. Repeated exposure to negative or dehumanizing content can influence users’ emotions (Utych, 2018) and perceptions (Hsueh et al., 2015; Del Vicario et al., 2016). For instance, a study analyzing Italian Facebook groups found a correlation between user activity and negative emotional states (Del Vicario et al., 2016). The study by Ahmed et al. (2021a) of a student sample in the Singaporean context reported that social media use was negatively related to anti-immigrant attitudes. Emerging evidence suggests that social media use relates to anti-immigrant attitudes. For instance, a recent study suggests a strong time-series correlation between social media consumption and offline hate crimes, as Donald Trump’s tweets on Islam-related topics were associated with a spurt in hate crimes during his presidency (Müller and Schwarz, 2023), while other research has also reported that online hate has offline spillovers in the form of hate crimes (Chan et al., 2016). These studies reinforce the role social media use, especially exposure to online prejudice, may play in subsequent user attitudes and behavior. However, it is essential to note that social media is more than just a platform for negativity. Research has highlighted its potential positive impact, such as fostering intergroup contact in post-conflict societies like Serbia, Croatia, Cyprus (Žeželj et al., 2017), and Israel (Walther et al., 2015). In the Singaporean context, our goal is to provide a comprehensive understanding of whether increased social media activity correlates with heightened anti-immigrant attitudes.

Next, utilizing the integrative threat theory (ITT) (Stephan and Stephan, 1996; Stephan and Stephan, 2000) as a foundation, we aimed to understand social media’s role in shaping anti-immigrant evaluations. ITT is a theoretical framework that explains the elements of individuals’ perceived threat contributing to social group prejudice. Two of the most critical threats in ITT are symbolic and realistic threats. Symbolic threats involve perceived challenges to a group’s cultural, social, or ideological identity, which are symbolic in nature and represent a potential change or shift that is perceived as endangering the values, norms, or worldview of a particular social group. Realistic threats involve more tangible risks that may pose immediate harm to a group or individual. These threats are grounded in observable and empirical phenomena, such as economic competition, territorial disputes, or physical harm (Stephan and Stephan, 2000). Therefore, we collected survey data from an online panel of Singaporeans, focusing on exposure to immigrant-related information and its subsequent relationship with prejudiced emotions toward immigrant groups. In our analysis, we explored the direct relationship between the use of social media and prejudiced emotions toward immigrant groups in Singapore. Building on ITT, we investigated whether symbolic and realistic threat perceptions mediate this relationship. Finally, we examined the role of cognitive ability in explaining the variance in the findings based on individual differences.

We offer an extension to the typical understanding of social media use and attitudes against outgroups (Hameleers and Schmuck, 2017; Davidson and Farquhar, 2020) to consider its association with *affective prejudice* – the broader set of prejudicial emotions evoked by immigrants (e.g., contempt, disgusted, angry, envious, and jealousy). Although most research discusses the relationship between threat perceptions and prejudice, several studies extend the discussion to focus on negative emotions against the outgroups. For example, some studies indicate that the two threat perceptions predict negative racial attitudes toward immigrant groups (Stephan et al., 2002, 2005). However, when analyzing the relationship between social media use and attitudes against outgroups, most scholars focus on prejudice (Hameleers and Schmuck, 2017; Davidson and Farquhar, 2020), failing to extend the discussion to broader emotions or attitudes such as affective prejudice (e.g., contempt, disgusted, angry, envious, and jealousy).

Recognizing the potential for varied perceptions of foreigners based on ethnicity, we grounded our selection of immigrant groups in both statistical data on the primary immigrant demographics in Singapore (Pan and Theseira, 2023) and prior research findings on outgroup prejudice within the nation (Chen et al., 2021; Ahmed et al., 2021a,b). For instance, Chen et al. (2021) found that Singaporean students exhibited differential warmth towards immigrant groups, with co-ethnic (Chinese) groups perceived differently from other ethnicities, while Americans were often perceived with greater warmth. Accordingly, we examined Singaporeans’ attitudes towards immigrants from both the major minority groups, such as Malays and Indians, as well as the Western or Caucasian immigrants as a broader category. Although the immigrant population in Singapore is diverse, with notable representations from countries like the United Kingdom, France, and Australia, we followed the convention Chen et al. (2021) adopted to term all Caucasian expats as “Americans.” Accordingly, we propose the following hypothesis:

*H1:* Social media use will be positively associated with negative emotions toward (a) American expatriates, (b) Malays, and (c) Indians.

In exploring the relationship between social media use and prejudice, it is also important to consider how *threat perception* can explain how social media exposure relates to negative emotions towards outgroups. The role of threat perception as a predictor of prejudice has been widely discussed (Stephan and Stephan, 1996; Stephan and Stephan, 2000; Esses et al., 2001). The Integrated Threat Theory (ITT) (Stephan and Stephan, 1996; Stephan and Stephan, 2000) model proposes four core components associated with outgroup prejudice. These are realistic threats, symbolic threats, negative stereotypes, and intergroup anxiety (Stephan and Stephan, 2000). Based on ITT’s original model, realistic and symbolic threats are most commonly discussed as predictors of prejudice (Stephan et al., 1999b). The realistic threats consist of threats to the ingroup’s existence, political and economic power, and physical well-being. Previous studies show that perceived realistic threat positively relates to prejudice towards outgroups (Quillian, 1995; Stephan et al., 1999b). Similarly, symbolic threat involves perceived group differences in values, norms, morals, or identity of the corresponding ingroup (Stephan et al., 2002). Prior studies also confirm the positive relationship between symbolic threat perception and prejudice (Sears, 1988; Stephan et al., 1999a).

Exposure to anti-immigrant content or discussions on social media can activate realistic and symbolic threat perceptions that are readily accessible in evaluating immigrant outgroups (as found in Study 1) (Mahmood et al., 2021). Previous research on Asian Americans in the United States also found that the stronger the social media users’ believe in the fairness and accuracy of the social media platforms that they use daily, the higher the likelihood that they will perceive Chinese pose a realistic and symbolic threat to the American (Croucher et al., 2020). Therefore, it is reasonable to expect that frequent use of social media will increase the salience of threat perceptions, which could help explain negative emotions toward immigrant outgroups. Furthermore, we expect a greater variance in effect sizes on immigrant attitudes, depending on the minority group under consideration, based on the prior findings from Chen et al. (2021). Hence, we propose the following hypotheses:

*H2:* Symbolic threat perception positively mediates the relationship between social media use and negative emotions toward (a) American expatriates, (b) Malays, and (c) Indians.

*H3:* Realistic threat perception positively mediates the relationship between social media use and negative emotions toward (a) American expatriates, (b) Malays, and (c) Indians.

Finally, we are also interested in individual factors such as cognitive ability – the higher-order mental processes that include reasoning, information processing, and problem-solving (Hodson and Dhont, 2015; Onraet et al., 2015) – in how individuals form attitudes. Cognitive ability reflects the mechanisms of how individuals learn, remember, solve problems, and pay attention. It reflects an interplay between different cognitive functions, offering a theoretical basis for anticipating how different cognitive abilities may moderate or influence other psychological processes or outcomes, such as beliefs and attitudes. Cognitive ability is essential to understand why some individuals may be more susceptible than others to online content (Bode, 2012): they may be more prone to believe what they read without critically evaluating it if they have low cognitive abilities (Pennycook and Rand, 2020).

In the context of this study, it is necessary to examine the relationship between cognitive ability and threat perception. Some prior work has accounted for cognitive ability in analyzing the factors predicting prejudice (Dhont and Hodson, 2014; Hodson and Dhont, 2015). Similarly, cognitive abilities are expected to influence individuals’ threat perceptions. For instance, individuals with lower cognitive ability tend to trust others less, are less sensitive to social cues, and are less accurate in perceiving others’ behaviors and intentions (Murphy and Hall, 2011). Higher levels of cognitive ability increase pro-social behavior, such as reducing the anchoring effect, improving altruism and levels of open-mindedness, and fostering more tolerant attitudes toward outgroups (Millet and Dewitte, 2007; Bergman et al., 2010). In contrast, lower levels of cognitive ability increase negative evaluation of outgroups by increasing negative emotional responses (i.e., anxiety, anger, disgust, fear) and negative attitudes (Hodson and Dhont, 2015; Utych, 2018; Croucher et al., 2020).

Prior research also suggests that individuals with lower cognitive ability are more likely to feel lower personal control over their circumstances, and therefore, they may be more likely to attribute agency to external factors (Lusk, 1983; Feather and Volkmer, 1988), such as the perceived threat from outgroups. Corroborating these expectations, experiments among university students have revealed that lower cognitive ability test scores predict higher perceived stereotype threat levels (Sawyer and Hollis-Sawyer, 2005). Other prior research has similarly reported that individuals with high information processing ability are less vulnerable to high-threat, negatively framed information and are better at rationally evaluating an object (Petty and Cacioppo, 1986; Igartua et al., 2012). However, to our knowledge, there is no prior work on how cognitive ability indirectly affects how threat perception mediates the relationship between social media use and anti-immigrant attitudes. Based on prior work, we expect that individuals with lower cognitive abilities who frequently use social media are more likely to perceive immigrants as threats (both symbolic and realistic), leading to stronger negative emotions toward the immigrant groups. On the other hand, the effect of threat perceptions may be weaker or non-existent for those with higher cognitive ability, where even if they use social media frequently, they are less likely to perceive immigrants as threats, resulting in weaker or no significant negative emotions toward the immigrant groups. Accordingly, we can test a moderated mediation relationship, positing that:

Cognitive ability moderates the mediated relationship between social media use and negative emotion toward (a) American expatriates, (b) Malays, and (c) Indians with the symbolic (H4) and realistic threat (H5), such that the mediated relationship is more robust for those with low cognitive ability than those with high cognitive ability.

**Figure 1:**
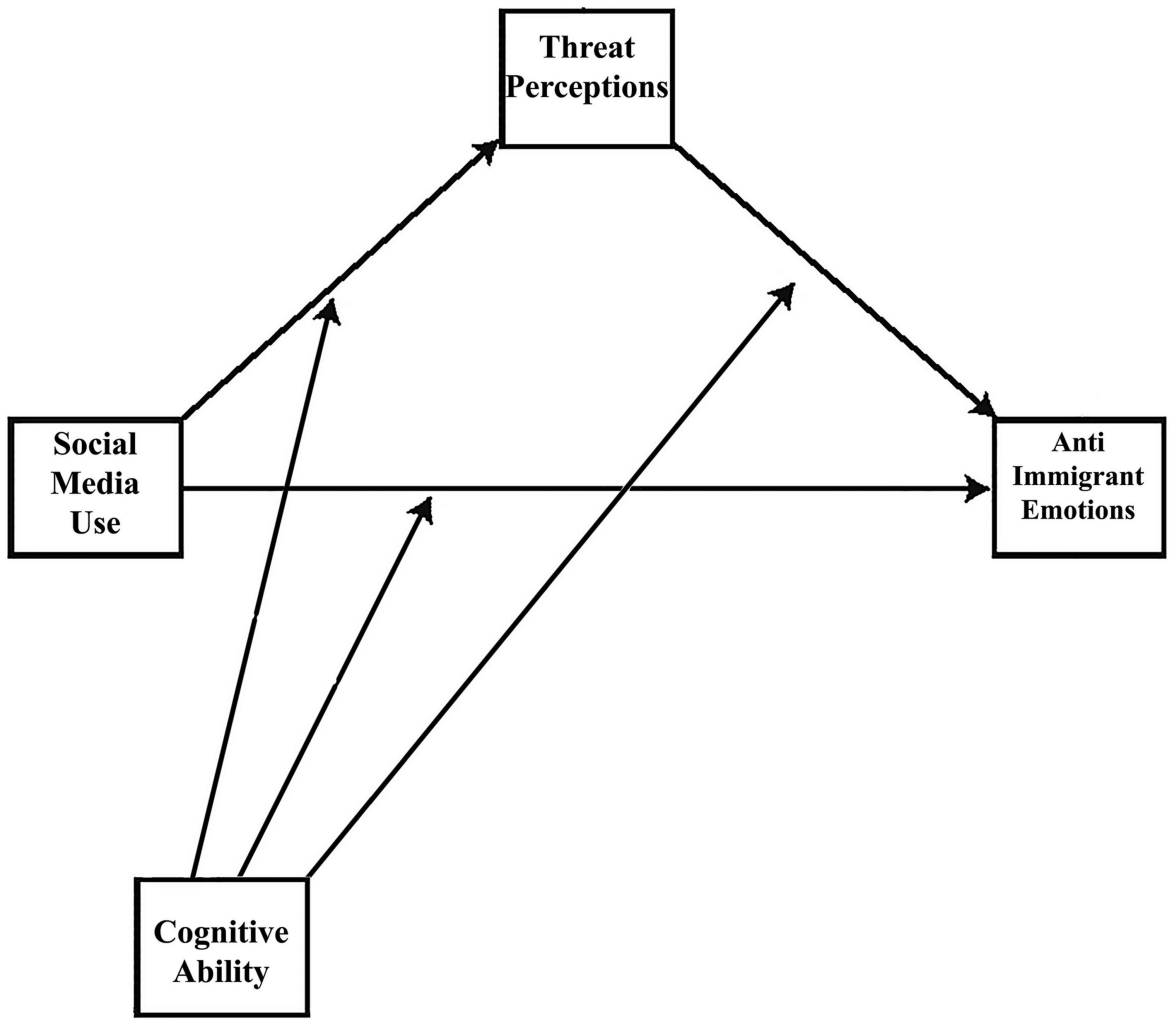
Conceptual framework.

The conceptual model in Figure 1 illustrates the various hypotheses.

To sum up, this study offers the opportunity to understand social media’s role in public opinion expression in Singapore, a non-liberal context with a complex social and ethnic hierarchy. We also build on prior work by considering and supplementing evidence of social media expression with social media use to better understand these relationships.

## 3 Study 1: a computational text analysis of Singaporean social media posts

The first aim of Study 1 is to identify the emotional tone with which immigrants are discussed in the public discourse on social media. We aim to do so by examining social media posts in Singaporean communities, where users express themselves and share personal thoughts (Macafee and De Simone, 2012). Analyzing social media posts offers an effective means for us to comprehend emotions and opinions on social media when discussing migrants. Second, we aim to investigate the contexts and connotations of immigrant mentions in popular public discourse in Singapore. Therefore, the data for Study 1 comprised social media discussions posted to internet communities and Facebook pages that are the most frequented by Singaporeans, and the approach involved the emotion analysis and topic modeling of the 86,462 filtered posts and comments about immigrants.

### 3.1 Data collection and pre-processing

In the first step, all the posts and comments for June 2018 to December 2018 were collected from internet communities that have the highest number of followers, as a representative proxy for the Singaporean online public sphere. Table 1 reports the number of followers for each community included in the data collection and the number of posts yielded from each source. Many Facebook community pages based out of Singapore were chosen, as over 70% of Singaporeans have a Facebook account (Statista, 2023). As seen in Table 1, the selection of Facebook pages comprises widely visited and followed community sites on a broad ideological spectrum (Mainstream Straits Time to the alternative Straits Times Review). The pages included one national newspaper (Straits Times), one broadcaster (Channel News Asia), five community sites (Stomp, Mothership, All Stuff Singapore, SMRT, MustShareNews, Reddit/Singapore), and three alternative news sites (TOC, Independent Sg, STR). Hardwarezone is an internet portal similar to Reddit, popular amongst Singaporeans, with different subcommunities that discuss aspects of Singaporean culture, with 700,000 registered members and 2 million unique visitors every month in 2018.

**Table 1:** The Facebook pages and Reddit community used to construct the Singapore Facebook dataset.

Source	Community name	Number of members	Number of posts	Number of English posts mentioning immigrants (>10 characters)
Facebook	Straits times review	50,389	115,249	21,127
	Channel news Asia	2,721,374	98,599	10,594
	Mothership	262,840	92,463	8,391
	Online citizen	105,350	76,211	14,041
	Straits time	1,260,745	47,272	4,618
	Independent SG	64,649	37,379	6,095
	Stomp	587,620	34,671	2,994
	All Singapore stuff	398,414	28,486	2,659
	SMRT	431,599	26,540	4,112
	Must share news	67,000	18,030	1,871
Hardwarezone.sg	Eat-drink-man-woman	NA	33,414	2,566
Reddit	/r/singapore	337,000	94,291	7,394
Total			488,924	86,462

Some statistics were not openly available from the sources and have been marked as NA (Not available).

Once the communities were chosen, data was collected using researcher APIs and Python code. In the case of Facebook, the Netvizz app (Rieder, 2013) accessed the Facebook Graph API and collected various types of data, including posts and comments. Next, discussion threads from Reddit and other forums were collected using the Python package Praw (Praw, 2020), which extracts data like posts, comments, and metadata from subreddits and user accounts. The data collection yielded 702,607 posts and comments (henceforth, we refer to the joint collection of posts and comments as “posts”).

In the second step, we identified those posts and comments that discussed immigrants. We used the Python natural language toolkit to retain only the posts and comments in English, thereby reducing the dataset to 488,924 posts and comments (henceforth referred to as “posts”). We then separated the dataset into those posts that mentioned immigrants versus those that did not by using a dictionary of 52 immigrant-related words, nouns, colloquial terms, and stereotypes from Singapore from prior work (Ahmed et al., 2021a). The dictionary comprises the lemmatized forms of these words. It includes general terms such as ‘migrant,’ ‘expat,’ (expatriate), certain visa statuses such as ‘CECA,’ ‘MIC,’ and ethnicities such as ‘Chinese,’ ‘PRC,’ ‘Ang Mo,’ Pinoy,’ and ‘FDW.’ The list also includes stereotypes and pejoratives such as “smellies” and “maids,” commonly used in Singapore to refer to working-class immigrants. In this manner, the final dataset mentioning immigrants comprised 105,539 English posts and comments, of which 86,462 posts were at least 10 characters. The final numbers of posts and comments from each source are reported in the last column of Table 1. The average length of a post was 13 words, and the median length was 11 words.

It is possible that some were posted by immigrants relating their personal experiences instead and are, therefore, “false positives” as they are irrelevant to our research objectives of analyzing attitudes towards immigrants. To validate our dataset, we constructed a random sample of 200 posts from the dataset of 86,462 filtered posts. We annotated them according to whether the post indicated either a personal experience of being an immigrant (irrelevant, therefore a false positive) or a comment about immigrants in the third person (relevant, therefore a true positive). A low false-positive rate (less than 1%) belays concerns that the dataset may inadvertently include immigrants’ perspectives. Therefore, we are confident that our dataset is appropriate for studying public discourse about immigrants in Singapore.

The following sections describe how we first conducted emotion analysis to understand public emotions towards immigrants. Emotion analysis involves examining, processing, summarizing, and reasoning about subjective texts infused with emotional tones. The proliferation of social media platforms has generated an extensive pool of emotional data, where emotion analysis techniques can be applied to unlock the meaning behind emotional texts (Chen, 2022). Next, we performed an automatic topic modeling approach to understand the major themes of discussions about immigrants in Singapore. Topic modeling is an approach that can autonomously assign relevant topics to each document based on their closest resemblance. It enables readers to quickly understand the overview of large documents (Sajid et al., 2017).

### 3.2 Measurements

*Negative emotion* was measured from the text by conducting an emotion analysis of the text of the discussions. The emotion analysis relied on a Singaporean English (Singlish) Senticnet lexicon (Ho et al., 2018), including emotion weights for Singaporean slang words. The analysis was also replicated with a different emotion measure from the Linguistic Inquiry and Word Count (LIWC; Pennebaker et al., 2015). LIWC has been frequently used to analyze linguistic properties in social media posts, including Singaporean social media posts (Tov et al., 2013). LIWC generates a continuous measure of the percentage proportion of positive and negative words in text.

*Topical themes* discussed in the text were identified through an unsupervised probabilistic topic modeling approach. First, stop words (common words which provide no useful information about topics) were removed. Additionally, words specifically referencing Singapore were excluded to ensure the identification of distinct inter-topic differences. However, pronouns, which are often instrumental in ingroup-outgroup analyses (Housley et al., 2010; Robertson et al., 2013), were retained. Subsequently, we adopted topic modeling techniques as suggested by prior research on Facebook and Twitter content (Schwartz and Ungar, 2015; Eichstaedt et al., 2018; Jaidka et al., 2021). Utilizing the Python Mallet package via the Differential Language Analysis Toolkit (DLATK), which is tailored for short social media posts (Schwartz et al., 2017), we conducted a grid search to determine the optimal alpha parameter and the number of topics. This process involved iterative reviews by the authors to ensure minimal word overlap across topics. An alpha value of 5 was selected, allowing the algorithm to detect up to five topics in lengthier posts, aligning with DLATK’s default recommendation for short posts. The final output consisted of 50 distinct topics, each represented by a set of words and their associated conditional probabilities, indicating the likelihood of a text (a social media post) belonging to a specific topic given the presence of a word.

### 3.3 Results

#### 3.3.1 The emotions expressed in the social media discourse about immigrants in Singapore

Comments with positive emotion, e.g., “Most Malaysians are hoping to see a better future (…)” expressed hope about immigrants, while posts labeled with negative emotions often reflected sarcasm, e.g., “(…) Fake Degree very handy for this FT,” or often compared races, e.g., “The Chinese are taking over smh (…).” We observed that sarcasm often complicated emotion measurement. For instance, the post “SG garmen just want to earn levies from FOREIGN trash from across. Cheaper and convenient…Useful or useless is secondary” reflected both positive and negative emotions towards immigrants.

Therefore, to contextualize measurements to the overall use of emotional words in the post, we designed a comparative analysis that compares the presence of negative and positive words between the immigrant discourse on social media and a referenced baseline of the general discourse on social media. This analytical design is more insightful in answering our research question and less susceptible to labeling errors when negative words are used, but a negative attitude may not be expressed.

We ran Welch Two Sample t-tests to compare the positive and negative emotion levels between the social media discussions mentioning immigrants and those that did not. The findings reported in Table 2 answer RQ1 and suggest that social media discussions involving immigrants are significantly less positive (*M* = 3.8%, *SE* = 5.6%) than general social media discussions (*M* = 5.8%, *SE* = 10.3%, *p* < 0.001). Posts mentioning immigrants also indicated significantly higher levels of negative emotion (*M* = 2.8%, *SE* = 4.4%) than general discussions (*M* = 2.6%, *SE* = 5.2%, *p* < 0.001). Reanalyzing the data with the LIWC emotion measure did not change the results, as discussions involving immigrants were significantly less positive (*M* = 3.7%, *SE* = 8.3%) and more negative (*M* = 2.2%, *SE* = 5.5%) than general discussions (*M* = 4.1%, *SE* = 11.1% for positive emotion, *p* < 0.001; *M* = 2.1%, *SE* = 6.9% for negative emotion, *p* < 0.001).

**Table 2:** Distribution of positive and negative emotions in posts about immigrants vs. posts about other topics.

		Posts about immigrants	Posts about other topics
		86,462	402,462
Positive emotion (%) Mean (Standard error)	Senticnet	3.8 (5.6)	5.8 (10.3)
	LIWC	3.7 (8.3)	4.1 (11.1)
Negative emotion (%) Mean (Standard error)	Senticnet	2.8 (4.4)	2.6 (5.2)
	LIWC	2.2 (5.5)	2.1 (6.9)

#### 3.3.2 The topical themes expressed in the social media discourse about immigrants in Singapore

Table 3 addresses RQ2, showcasing the topics discerned through this probabilistic topic modeling of social media discussions about immigrants. Each topic is characterized by its most significant words, ranked by importance. The topic modeling approach we employed is inherently algorithmic and automated. As such, the generation of topics was not subject to human interpretation or bias, eliminating the need for inter-rater reliability assessments. However, to streamline the presentation of results, topics with similar themes, as determined by overlapping frequent words, were grouped for a more coherent illustration.

**Table 3:** Some of the main concerns generated through a topic modeling approach on the posts mentioning immigrants.

Theme	Word clouds		
Self-reference 15.1%	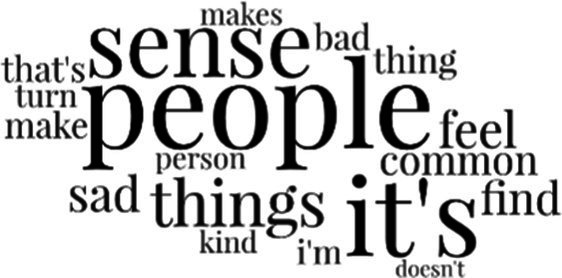	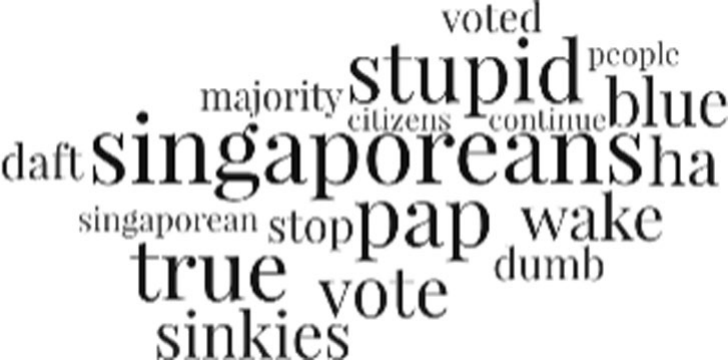	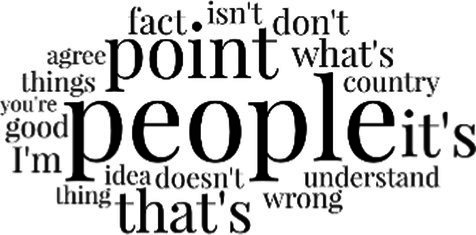
Government 12.7%	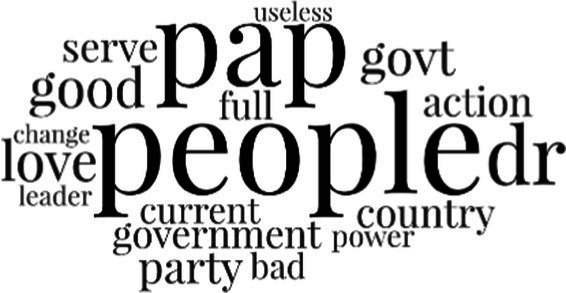	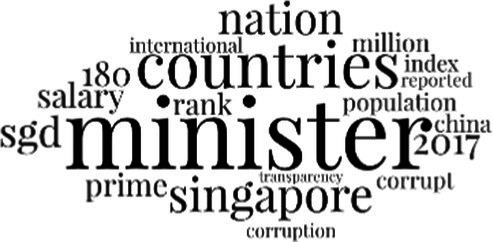	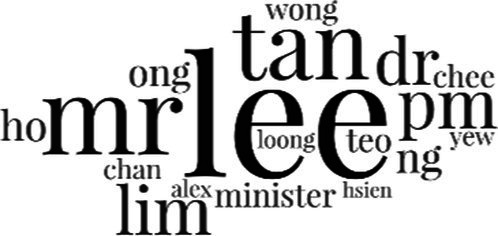
Jobs 11.6%	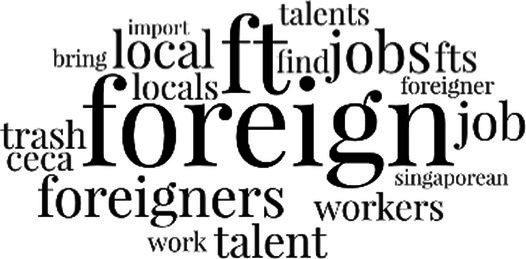	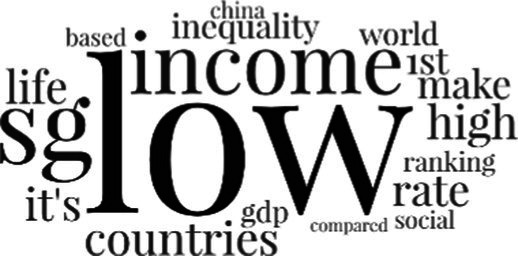	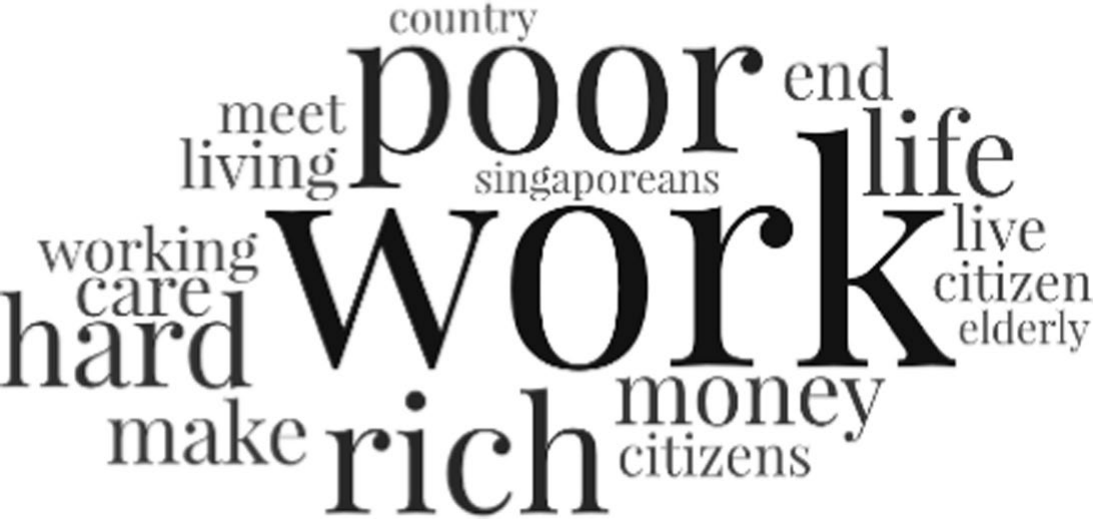
Economy 4.5%	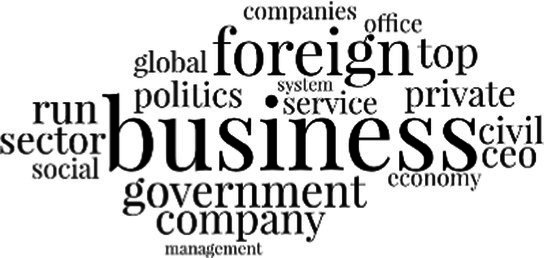	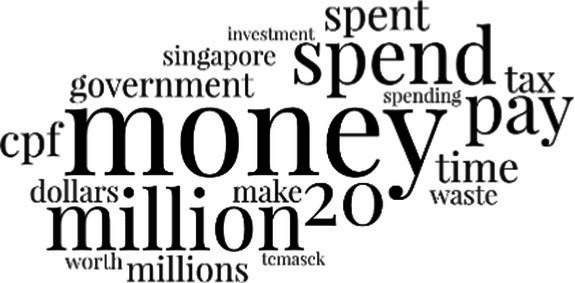	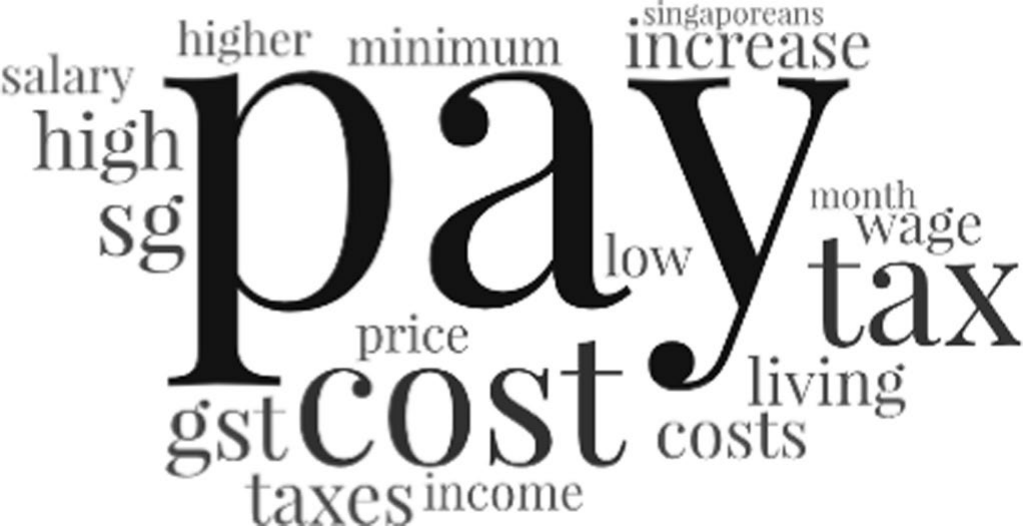
Crime and law 6.9%	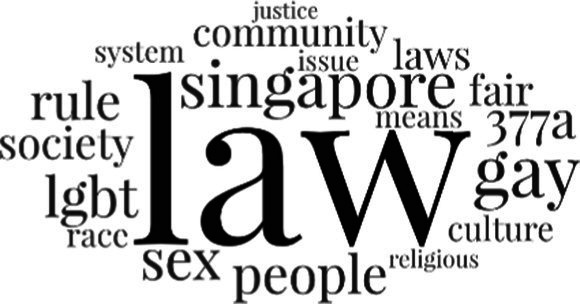	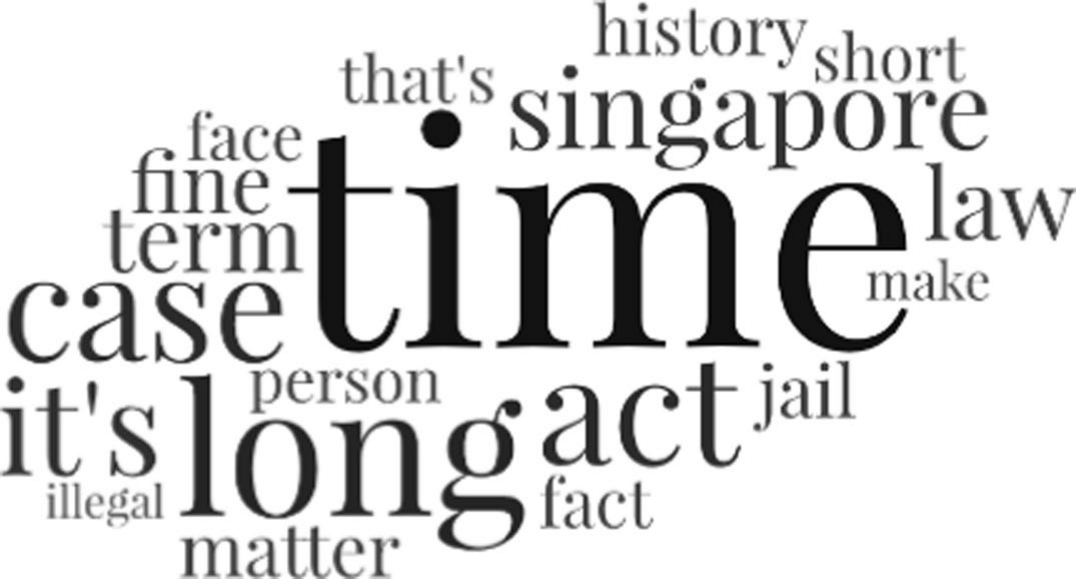	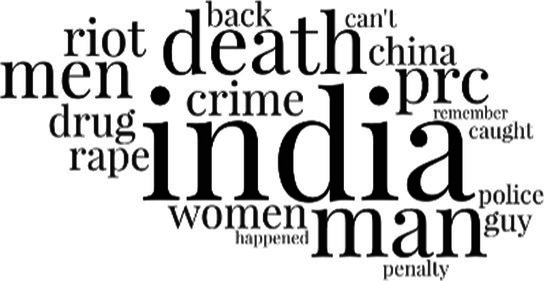
Security 2.3%	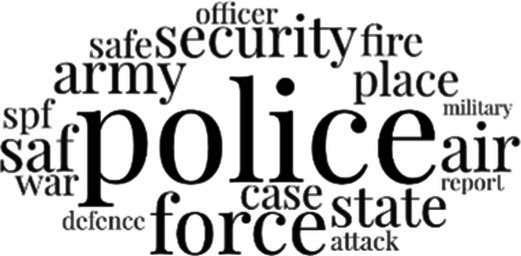		
Habits and culture 4.02%	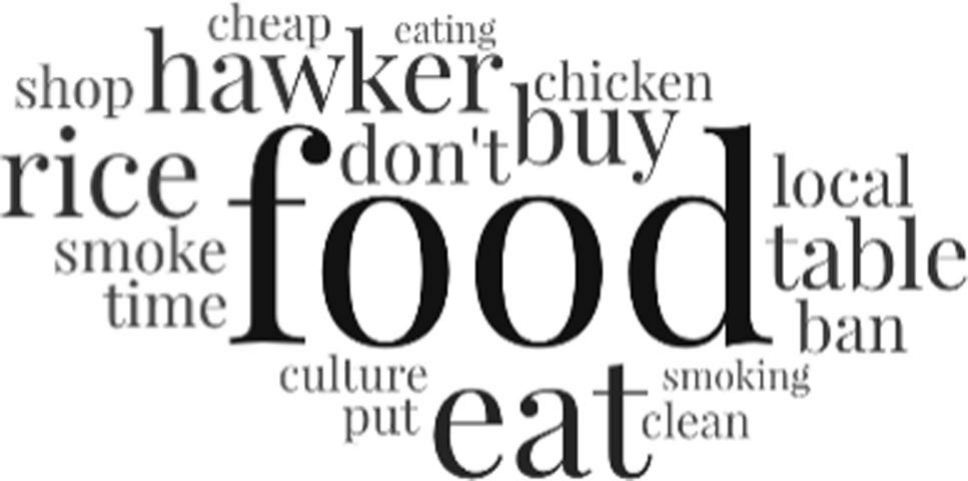	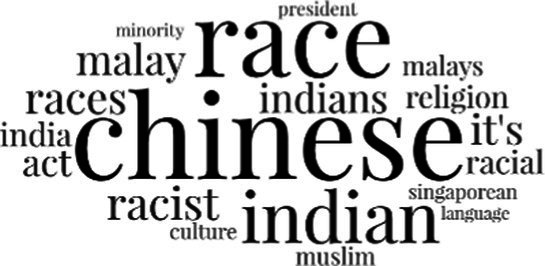	

Latent Dirichlet allocation was used to generate 50 topics mentioning immigrants in social media posts. *α* = 5 favored more topics per post.

A notable observation is that over 15% of posts discussing immigrants also reference Singaporeans, implying a comparative stance: “Telling you Singaporeans not working hard enough while their favorite foreign trash (…).” The government emerges as another dominant context in these discussions, accounting for 12.7% of all posts, e.g., “This has nothing at all to do with what party is in control of the government.” The discourse predominantly concerns immigrants’ economic impact, emphasizing jobs (11.6%), e.g., “(Foreign talents) gets the good jobs and pulls down productivity (…)” and the economy (4.5%), e.g., “Our rice bowl is always threatened by others who will work for cheaper and will work longer hours!.” Other discussions highlight concerns related to national security (2.3%) and legal implications (6.9%). Conversely, a smaller proportion of the discourse delves into foreigners’ cultural habits (4.02%).

#### 3.3.3 Discussion

Study 1’s findings yield two pivotal insights that underscore the need for a deeper exploration of inter-group dynamics in Singapore. The thematic exploration revealed a perception of immigrants as potential threats to Singapore’s culture and economic equilibrium. Overall, the pronounced hostility towards immigrants in the online discussions suggests a potential undercurrent of discontent or prejudice within the local populace.

These insights from Study 1 catalyzed the conception of Study 2, aiming to delve deeper into the threat dimension of anti-immigrant emotions in Singapore at a more granular, individual level. Study 2 was designed as a national survey targeting Singaporeans. This survey approach aimed to uncover the root causes of anti-immigrant emotions. By transitioning from a broad observational lens to a more targeted survey methodology, we hoped to enrich our understanding of public discourse. This would allow us to pinpoint both the triggers and outcomes of specific behaviors and their varied impacts across different segments of social media users. For instance, some users are influenced more by their peers (Bode, 2012), or those with lower cognitive abilities might be more susceptible to online misinformation (Pennycook and Rand, 2020), making them potentially more vulnerable to the narratives they encounter online.

## 4 Study 2: a national survey of Singaporean adults

Study 1 identified a few perceived threats to foreigners in Singapore based on a computational text analysis of the expressive use of social media. To complement this approach, Study 2 focuses on the informational use of social media and its association with audience opinions. It uses the lens of integrated threat theory to empirically examine the psychological mechanisms underlying the association of social media consumption (not expression) with prejudiced emotions against immigrants. We report the findings based on an analysis of data from a national survey, focusing on the relationships between social media use, threat perceptions, cognitive ability, and negative emotions toward immigrant outgroups. Survey responses were collected in February 2020 from an online panel managed by Qualtrics. The sample was closely matched to the population parameters, focusing on age and gender to increase the generalizability.

According to the Department of Statistics Singapore (2016), there were around 4 million Singaporean citizens and permanent residents. Based on prior literature on the mediation of user attitudes through beliefs (Lee et al., 2023), we anticipate small effect sizes (<0.05) for the indirect effects; therefore, to obtain a margin of error of 4% and a confidence interval of 95% for the main study, a power analysis suggested that 857 valid responses will be needed. Therefore, the final sample included responses from 1,036 Singaporean citizens to account for possible issues in data quality.

### 4.1 Measures

The descriptive details of the main variables of interest are included in Table 4.

**Table 4:** Descriptive and reliability of primary variables of interest.

	American expatriates		Malays		Indians	
Variable	*M* (SD)	*α*	*M* (SD)	*α*	*M* (SD)	*α*
Negative emotions	3.00 (1.45)	0.92	2.78 (1.38)	0.91	2.99 (1.44)	0.90
Symbolic threat	2.48 (0.98)	0.92	2.28 (1.01)	0.95	2.77 (1.13)	0.95
Realistic threat	2.50 (0.92)	0.89	2.24 (0.97)	0.95	2.90 (1.09)	0.93
Social media use	*M* = 3.46 SD = 1.57 Cronbach’s *α* = 0.89					
Cognitive ability	*M* = 5.27 SD = 1.98 Cronbach’s *α* = 0.73					

*Negative emotions* against immigrant groups were measured by asking the respondents, in general, how they feel toward (each immigrant group) in Singapore across five dimensions: (a) contempt, (b) disgust, (c) anger, (d) envious, and (e) jealousy (see Cuddy et al., 2008). The responses measured on a 7-point scale (1 = not at all to 7 = a lot) were averaged to create a scale of negative emotions.

*Social media use* was measured by asking the respondents how frequently (1 = never to 7 = several times a day) they use the following features of social media for information and news purposes related to political and social issues: (a) post in their timeline (b) comment on posts (c) share posts (d) read their newsfeed (e) read their friends’ timeline (f) search with the search bar on their social media homepage. The responses were averaged to create a scale of social media use.

In the Singaporean context, the *symbolic threats* measure was adapted from previous work (Tausch et al., 2007; Rupar and Graf, 2019). Respondents were asked to rate their level of agreement with the following statements (a) Singapore’s identity is being threatened because there are too many (immigrant groups) in Singapore; (b) Singaporean norms and values are being threatened because of the presence of (immigrant groups) in Singapore and (c) (Immigrant group) in Singapore are a threat to the Singaporean culture. The items measured on a 5-point scale (1 = strongly disagree to 5 = strongly agree) were averaged to create a scale of symbolic threat.

The *realistic threats* measure was also adapted from previous work (Tausch et al., 2007; Rupar and Graf, 2019) to the Singaporean context. Respondents were asked to rate their level of agreement for four statements: Because of the presence of (immigrant group) (a) Singapore is overcrowded, (b) Singaporeans have difficulties finding a job, (c) Singaporeans have more difficulties finding a house, and (d) Singapore is getting dirty. The items measured on a 5-point scale (1 = strongly disagree to 5 = strongly agree) were averaged to create a scale of realistic threat.

*Cognitive ability* was measured by a frequently used 8-item WordSum test where participants were asked to match a given word to the closest matching word from a target list of five choices. The specific vocabulary test shares high variance with general intelligence and has been consistently used to measure the cognitive ability of respondents (Colom et al., 2005; Brandt and Crawford, 2016; Ganzach et al., 2019) as well as in recent studies related to information consumption and attitude formation (Pennycook and Rand, 2020, 2021). The test is also valid for the Singaporean sample since English is the primary language of the educational system and the most spoken language (Department of Statistics Singapore, 2016).

*Demographic variables* included age (*M* = 40.61, SD = 12.91), gender (52.3% males), education (1 = no formal education to 9 = post-graduate degree, Median = college under-graduate degree), income (1 = less than $1,000 to 11 = more than $20,000; Median = $7,000 to $8,999), race (82% Chinese, majority), and religion (33% Christianity, majority).

*Media news use* variables asked respondents how frequently (1 = never to 7 = several times a day) they use (a) television (*M* = 4.72, *SD* = 1.84), (b) radio news (*M* = 3.97, *SD* = 2.07) and (c) print newspaper (*M* = 3.98, *SD* = 1.96) to get information about political and social issues.

### 4.2 Results

#### 4.2.1 The informational use of social media and anti-immigrant attitudes

The study employed an ordinary least squares regression to predict negative emotions toward the three immigrant groups (see Table 5). H1 is supported as social media use was positively associated with negative emotions toward all three groups: American expatriates (*β* = 0.132, *p* < 0.001), Malays (*β* = 0.153, *p* < 0.001), and Indian (*β* = 0.122, *p* < 0.001) immigrants. On the other hand, those with higher use of traditional media such as television (American expatriates: *β* = −0.148, *p* < 0.001; Malays: *β* = −0.100, *p* < 0.001, and Indians: *β* = −0.151, *p* < 0.001) are less likely to hold negative emotions.

**Table 5:** OLS hierarchical regression predicting negative emotions against immigrant groups.

American expatriates		Malays	Indians
Block 1: demographics			
Age	−0.088**	−0.100***	−0.034
Gender (Females)	−0.024	−0.006	−0.050
Education	−0.005	−0.057	−0.019
Income	−0.018	−0.004	−0.008
Race (reference = Chinese)			
Malay	−0.031	0.012	−0.066
Indian	−0.061	−0.049	−0.069
Eurasian	−0.058*	−0.038	−0.044
Others	−0.036	−0.017	−0.059
Religion (reference = Christianity)			
Buddhism	−0.023	−0.028	−0.040
Hinduism	0.032	0.004	0.036
Islam	0.064	−0.026	0.050
Taoism	0.021**	0.001	0.028
Free-thinker	−0.011	−0.038	−0.039
Agnostic	−0.033	−0.047	−0.023
Atheist	−0.075*	−0.043	−0.061
Others	0.020	0.014	0.038
*R*^2^ change (%)	4.1	3.1	3.5
Block 2: Media use			
Television	−0.148***	−0.100**	−0.151***
Radio	0.175***	0.130***	0.172***
Newspaper	−0.021	0.006**	−0.025
Social media	0.132***	0.153***	0.122***
*R*^2^ change (%)	4.3	3.8	4.0
Block 3: Mediators (threat perception)			
Symbolic threat	0.220**	0.279***	0.304***
Realistic threat	0.295***	0.366***	246 **
*R*^2^ change (%)	22.2	35.4	25.8
Block 4: moderated-mediation factor			
Cognitive ability	−0.091***	−0.125***	−0.159***
*R*^2^ change (%)	0.70	1.2	2.1
Total *R*^2^ (%)	31.3	43.6	35.5

^*^p < 0.05.

^**^p < 0.01.

^***^p < 0.001.

Block 1 also includes contextual control in COVID-19 threat perception. As detailed in Section 4.1, the race and religion reported the majority of the respondents were chosen as the reference (82% Chinese; 33% Christianity).

#### 4.2.2 Symbolic and realistic threats mediate the association of social media use and anti-immigrant attitudes

First, considering the direct relationships reported in Table 5, both symbolic (American expatriates: *β* = 0.220, *p* < 0.001; Malays: *β* = 0.279, *p* < 0.001, and Indians: *β* = 0.304, *p* < 0.001) and realistic threat perception (American expatriates: *β* = 0.295, *p* < 0.001; Malays: *β* = 0.366, *p* < 0.001, and Indians: *β* = 0.246, *p* < 0.001) were positively associated with negative emotions toward all three immigrant outgroups.

**Figure 2:**
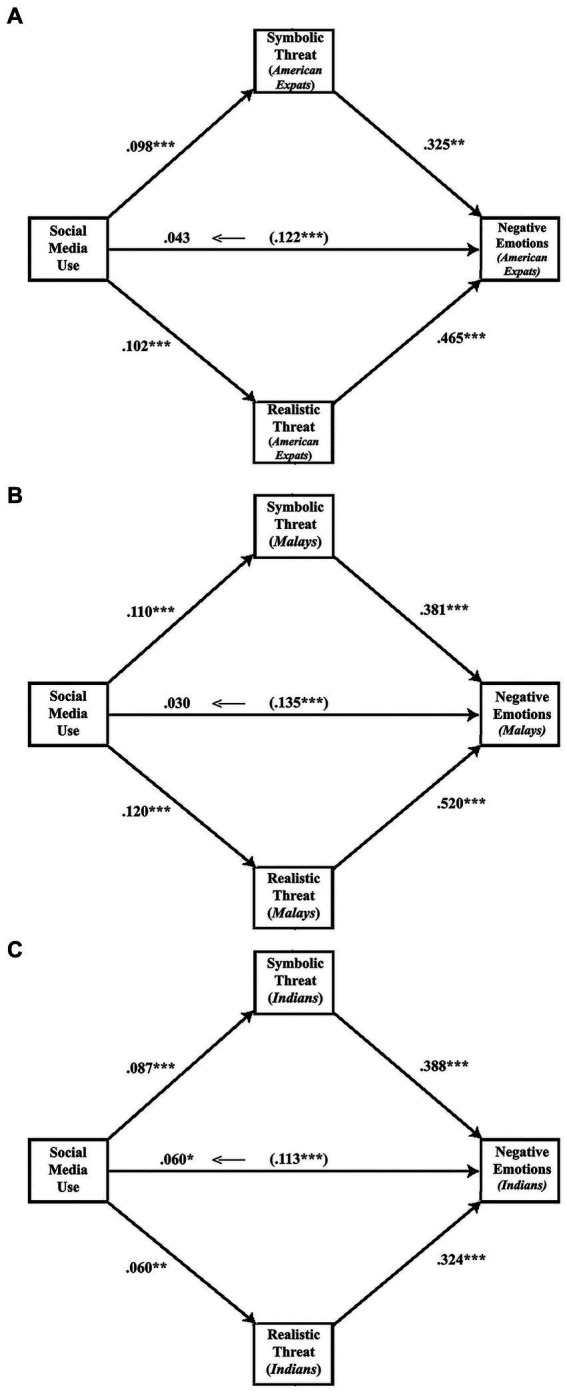
Illustration of the mediation analysis for the three outgroups.

Hypotheses 2 and 3 predict that symbolic threats (H2a-c) and realistic threats (H3a-c) mediate the relationship between social media use and negative emotions. We employed the SPSS macro PROCESS (Hayes, 2017). We employed a parallel multiple mediation model (Model 4) with bootstrapping to estimate the indirect mediation effects. The relationships between social media use, symbolic threat perception, realistic threat perception, and negative emotions toward immigrant groups are illustrated in Figure 2.

First, the results in Figure 2 suggest that those who frequently use social media are more likely to consider immigrant groups as both symbolic (Figure 2A American expatriates: *b* = 0.098, *se* = 0.023, *p* < 0.001; Figure 2B Malays: *b* = 0.110, *se* = 0.023, *p* < 0.001, and Figure 2C Indians: *b* = 0.087, *se* = 0.026, *p* < 0.001) and realistic threats (Figure 2A American expatriates: *b* = 0.102, *se* = 0.021, *p* < 0.001; Figure 2B Malays: *b* = 0.120, *se* = 0.022, *p* < 0.001, and Figure 2C Indians: *b* = 0.060, *se* = 0.025, *p* < 0.001).

Next, across all models, both symbolic (Figure 2A American expatriates: *b* = 0.325, *se* = 0.068, *p* < 0.001; Figure 2B Malays: *b* = 0.381, *se* = 0.068, *p* < 0.001, and Figure 2C Indians: *b* = 0.388, *se* = 0.064, *p* < 0.001) and realistic threats (Figure 2A American expatriates: *b* = 0.465, *se* = 0.072, *p* < 0.001; Figure 2B Malays: *b* = 0.520, *se* = 0.071, *p* < 0.001, and Figure 2C Indians: *b* = 0.324, *se* = 0.067, *p* < 0.001) were positively associated with negative emotions.

Finally, Table 6 presents the parallel mediation results comparing the two paths of indirect effects: (i) social media use – symbolic threat – negative emotions, (ii) social media – realistic threat – negative emotions.

**Table 6:** Mediation of social media use through threat perceptions predicting negative emotions against immigrant groups.

Predicting negative emotions toward American expatriates				
Effects	*B*	SE	95% CI	
Total	0.122	0.033	0.057	0.187
Direct	0.043	0.029	−0.015	0.101
Indirect	0.079	0.018	0.046	0.117
SM SYMT NEMO	0.032	0.011	0.013	0.055
SM RELT NEMO	0.047	0.013	0.025	0.077

Predicting negative emotions toward Malays				
Effects	*B*	SE	95% CI	
Total	0.135	0.032	0.072	0.197
Direct	0.030	0.026	−0.020	0.080
Indirect	0.105	0.022	0.063	0.147
SM SYMT NEMO	0.042	0.013	0.019	0.070
SM RELT NEMO	0.063	0.016	0.035	0.096

Predicting negative emotions toward Indians				
Effects	*B*	SE	95% CI	
Total	0.113	0.033	0.048	0.179
Direct	0.060	0.029	0.004	0.116
Indirect	0.053	0.019	0.018	0.092
SM SYMT NEMO	0.034	0.012	0.012	0.061
SM RELT NEMO	0.019	0.010	0.003	0.042

1. Analyses performed using the PROCESS macro for SPSS (Model 4; Hayes, 2017) applying 5,000 bootstrapped bias-corrected resample 2. *B*, unstandardized coefficient; SE, standard error; CI, confidence interval; SM, social media use; SYMT, symbolic threat; RELT, realistic threat; NEMO, negative emotions. 3. Statistical controls include age, gender, education, income, race, religion, media use, and contextual control variable.

Now, consider the results for each immigrant group, reported in Table 6. First, for negative emotions toward American expatriates, the role of both symbolic threat (*b* = 0.032, *SE* = 0.011, 95% *CI* = 0.013 to 0.055) and realistic threat (*b* = 0.047, *SE* = 0.013, 95% *CI* = 0.025 to 0.077) as mediators between social media use and negative emotions was statistically significant. The direct effect was insignificant (*b* = 0.043, *SE* = 0.029, 95% *CI* = −0.015 to 0.101). Thus, H2a and H3a are supported.

Next, for negative emotions toward Malay immigrants, both symbolic (*b* = 0.042, *SE* = 0.013, 95% *CI* = 0.019 to 0.070) and realistic threats (*b* = 0.063, *SE* = 0.016, 95% *CI* = 0.035 to 0.096) were found to be statistically significant mediators. Similar to previous cases, direct effects were found to be statistically insignificant (*b* = 0.030, *SE* = 0.026, 95% *CI* = −0.020 to 0.080). Since indirect effects are statistically significant (*b* = 0.105, *SE* = 0.022, 95% *CI* = 0.063 to 0.147), H2b and H3b are supported.

Finally, for negative emotions toward Indian immigrants, the role of both symbolic (*b* = 0.034, *SE* = 0.012, 95% *CI* = 0.012 to 0.061) and realistic threat (*b* = 0.019, *SE* = 0.010, 95% *CI* = 0.003 to 0.042) as mediators between social media use and negative emotion was found to be statistically significant. Direct effects were also statistically significant (*b* = 0.060, *SE* = 0.029, 95% *CI* = 0.004 to 0.116). Since the indirect effects are significant, H2c and H3c are supported.

To sum up, the parallel multiple mediation analyses suggest that the effects of social media use on negative emotions toward the three immigrant groups are significantly mediated through symbolic and realistic threat perceptions; however, the directionality of the relationship is uncertain, as greater negative emotions may also reinforce more social media use because of higher symbolic and realistic threat perceptions.

#### 4.2.3 Cognitive ability moderates the association of threats with anti-immigrant attitudes

The direct relationships reported in Table 5 indicate that individuals with higher cognitive ability were less likely to feel negatively toward the immigrant groups (American expatriates: *β* = −0.091, *p* < 0.001; Malays: *β* = −0.125, *p* < 0.001, and Indians: *β* = −0.159, *p* < 0.001).

The next step was to test the final set of hypotheses (H4a-c and H5a-c), which predicted that cognitive ability would moderate the mediation of threat perception (symbolic and realistic) on social media. Again, we employed a conditional PROCESS (Model 59). Table 7 presents the indirect effect estimates, standard errors, and the 95% confidence interval significance value of moderated mediations. The indirect effects are estimated at three levels of cognitive ability: one standard deviation below the mean, the mean, and one standard deviation above the mean.

**Table 7:** Moderated mediation results for negative emotions against immigrant groups through threat perceptions as mediators and cognitive ability as a moderator.

Indirect effects in predicting negative emotions toward American expatriates				
Mediator	Cognitive ability	Indirect effects	SE	95% CI
Symbolic threat	3.276	0.032	0.014	0.008 to 0.063
	5.263	0.018	0.009	0.003 to 0.037
	7.251	0.005	0.011	−0.015 to 0.030
Realistic threat	3.276	0.050	0.019	0.019 to 0.093
	5.263	0.031	0.012	0.010 to 0.056
	7.251	0.009	0.017	−0.023 to 0.041

Indirect effects in predicting negative emotions toward Malays				
Mediator	Cognitive ability	Indirect effects	SE	95% CI
Symbolic threat	3.276	0.030	0.018	0.005 to 0.074
	5.263	0.023	0.011	0.005 to 0.046
	7.251	0.010	0.015	−0.019 to 0.040
Realistic threat	3.276	0.072	0.025	0.029 to 0.126
	5.263	0.039	0.014	0.015 to 0.069
	7.251	0.014	0.014	−0.012 to 0.044

Indirect effects in predicting negative emotions toward Indians				
Mediator	Cognitive ability	Indirect effects	SE	95% CI
Symbolic threat	3.276	0.029	0.016	0.005 to 0.068
	5.263	0.027	0.012	0.006 to 0.052
	7.251	0.023	0.017	−0.008 to 0.060
Realistic threat	3.276	0.035	0.018	0.004 to 0.076
	5.263	0.019	0.011	0.001 to 0.043
	7.251	0.007	0.011	−0.011 to 0.033

1. Analyses performed using the PROCESS macro for SPSS (Model 59; Hayes, 2017) applying 5,000 bootstrapped bias-corrected resample 2. SE, standard error; CI, confidence interval 3. Cognitive ability, -1SD, mean, and + 1SD 4. Statistical controls include age, gender, education, income, race, religion, media use, and contextual control variable.

Moderated mediation is observed when the strength of an indirect effect depends on the moderator’s levels (Preacher and Hayes, 2004). As observed across all models, the indirect effects of symbolic threats significantly decreased as the level of cognitive ability increased. It was stronger for individuals with lower cognitive ability (American expatriates: *b* = 0.032, 95% *CI* = 0.008 to 0.063, Malays: *b* = 0.030, 95% *CI* = 0.005 to 0.074; Indians: *b* = 0.029, 95% *CI* = 0.005 to 0.068) than average cognitive ability individuals (American expatriates: *b* = 0.018, 95% *CI* = 0.003 to 0.037, Malays: *b* = 0.023, 95% *CI* = 0.005 to 0.046; Indians: *b* = 0.027, 95% *CI* = 0.006 to 0.052). The indirect effects for individuals with high cognitive ability are statistically insignificant.

A similar pattern is observed for the indirect effects of realistic threats, where the effects significantly decrease as the level of cognitive ability increases. The effects were stronger for individuals with lower cognitive ability (American expatriates: *b* = 0.050, 95% *CI* = 0.019 to 0.093, Malays: *b* = 0.072, 95% *CI* = 0.029 to 0.126; Indians: *b* = 0.035, 95% *CI* = 0.004 to 0.076) than average cognitive individuals (American expatriates: *b* = 0.031, 95% *CI* = 0.010 to 0.056, Malays: *b* = 0.039, 95% *CI* = 0.015 to 0.069; Indians: *b* = 0.019, 95% *CI* = 0.011 to 0.043). The indirect effects of high cognitive ability individuals were not statistically significant in any of the models.

These results suggest that cognitive ability moderates the relationship between threats and negative emotions toward immigrants such that the effects are stronger for individuals with low or average cognitive ability; however, as before, the directionality of the relationship cannot be deterministically ascertained. Thus, H4a and H5a (American expatriates), H4b and H5b (Malays), and H4c and H5c (Indians) are supported.

### 4.3 Discussion

Study 2 drilled down to an individual-level analysis of the drivers of negative emotion towards immigrants, focusing on threat perceptions (realistic and symbolic threats) and cognitive ability. The findings suggest that individuals with higher informational use of social media and higher symbolic and realistic threat perception are more likely to exhibit anti-immigrant emotions. Symbolic and realistic threat perceptions positively mediated the relationship between social media use and anti-immigrant emotions. Furthermore, individuals with low cognitive ability are more likely to have negative emotions toward immigrant groups from gaining threat perceptions while using social media than those with average or high cognitive ability.

The findings are steady across three immigrant groups, highlighting that cognitive ability is a robust predictor of anti-immigrant attitudes. Further, the study confirms and expands existing research, explicating the direct associations. Finally, it shows that cognitive ability can directly impact prejudice and act as a contingency factor in how users evaluate sociopolitical content on social media, leading to prejudiced emotions.

## 5 General discussion

Social media may become a crucial outlet for citizens to voice concerns and opinions. The findings presented here suggest that social media discussions about immigrants generally adopt a negative tone and touch upon the themes of the economic and cultural threats perceived by the local community. The survey findings highlight that cognitive ability is a robust predictor of anti-immigrant affect, and they are stable across different immigrant groups. Finally, we show that a lack of cognitive ability is directly associated with higher prejudice; it also acts as a contingency factor in how users evaluate sociopolitical content on social media, and is associated with higher negative emotions.

There are three main takeaways from this work. The first takeaway underscores social media’s potential to qualitatively gauge public opinion, even in non-liberal contexts with limited press freedom. By analyzing aggregate emotional and topical signals from thousands of public posts, we can derive theoretically valid insights into the priorities and concerns of a citizenry. This real-time monitoring of public emotion can be instrumental in identifying unexpected surges in discussions, allowing for timely interventions to prevent potential hate crime incidents. For instance, recent acts of hate and extremism in France (Forbes, 2016), New Zealand (Cable News Network (CNN), 2019), and the United States (New York Times, 2021) were premeditated on social media before manifesting as real-life tragedies. The utility of social media as a live emotion monitor lies potentially in the immediacy and widespread reach of social media platforms that might amplify particular emotions, leading to rapid mobilization around specific issues or ideologies (Stephan and Stephan, 2000). By continuously monitoring these platforms, policymakers can proactively address emerging concerns through targeted public and social messaging efforts (Esses et al., 2001).

The second takeaway emphasizes the significance of inter-group differences when exploring social media’s influence on attitude formation. Our findings indicate that informational use of social media correlates positively with negative emotions towards all three outgroups – Indians, Americans, and Malays. Individual differences in perceptions of realistic and symbolic threats were pivotal in mediating this relationship.

The mediation relationship can be explained based on the broader political opinion literature on the role of individual differences. Echo chambers, which can arise due to homophily or contagion, might reinforce existing biases, intensifying threat perceptions against immigrants (O’Hara and Stevens, 2015). However, the causality direction remains uncertain. Alternatively, these findings might stem from individual differences rooted in self-interest, self- and social categorization, and individual social identities (Sears and Funk, 1991; David, 2009; Bachl, 2017). This suggests a complex interplay between social media exposure, individual predispositions, and the formation of prejudiced attitudes. Furthermore, it is indeed possible that more prejudiced media users interpret social media content through a biased lens. Our study does not refute this; instead, it attempts to investigate whether the specific nature of social media engagement can compound or alter these perceptions. In combining our two studies, we aim to provide a more comprehensive understanding of the interplay between media use, individual predispositions, and perceptions toward immigrants. To further refine our understanding, future research could delve deeper into the types of social media content consumed and the context in which social media interactions occur. This would help distinguish the media effects from the individual attribute effects more clearly.

The third takeaway reveals that social media users with low cognitive ability are particularly susceptible to the detrimental effects of social media, exhibiting higher levels of prejudice. Our data suggests a negative association between cognitive ability and anti-immigrant affect across all three immigrant groups. Moreover, cognitive ability was found to moderate the mediation effect via threat perceptions for all these groups. Therefore, the role of realistic and symbolic threats in tuning social media use into prejudiced emotions is more significant in individuals with lower and moderate cognitive ability levels than in individuals with high cognitive ability.

The study confirms existing research explicating the direct associations and expands on it, showing that cognitive ability might influence how individuals process and evaluate information, especially sociopolitical content, on social media platforms (Pennycook and Rand, 2020). Those with lower cognitive abilities might be more prone to accepting misinformation or being influenced by biased narratives, leading to heightened prejudiced emotions (Ahmed et al., 2021a,b). This aligns with existing research that has explored direct associations between cognitive ability and prejudice, suggesting that cognitive ability can influence both direct prejudice and how users interpret sociopolitical content, culminating in prejudiced emotions.

Our studies have a few limitations, which we attempted to offset through a multi-method analysis. For Study 1, first, despite meticulous efforts to curate data from local Singaporean platforms and validate its authenticity, there remains an inherent uncertainty about the nationality of all contributors. This potential mix could influence the overall tenor of the findings derived solely from social media data. Second, by its nature, social media might amplify the voices of a vocal minority, potentially overshadowing the more nuanced emotions of the silent majority. Third, while the observational nature of social media data can spotlight the prevailing negative rhetoric about immigrants, it offers limited insights into the impact of such rhetoric on its audience. Finally, aggregate trends from social media analysis can not delve into individual variances in immigrant perceptions or elucidate the personal traits and mechanisms driving these attitudes.

Although a multi-methodological framework combining social media and survey data increases the reliability of the findings, the study has several limitations that need to be discussed. First, while we anticipate that social media collected from popular internet communities would offer an unfiltered and unsolicited insight into attitudes towards immigrants, it is also true that relying on these posts alone is prone to reflect the most extreme views rather than those of the general public (Bail, 2021). Therefore, it is crucial to vet social media-based findings against survey-based results. Second, analyzing a multilingual sample also has its limitations. We restricted our analysis to include only English posts because of the lack of resources to translate other posts. While most Singaporeans express themselves in English on social media, we still lose out on discourse in other local languages and, therefore, by minority populations (e.g., Tamils and Malays). Finally, regarding the survey component of the analysis, while high threat perceptions and low cognitive ability are associated with heightened anti-immigrant feelings in response to social media use, a causal claim cannot be tested without a longitudinal research design.

## 6 Conclusion

The principal goal of this study was to compare the signals available through the expressive and informational use of social media in terms of their ability to explain local attitudes towards immigrants in Singapore. Therefore, this study conducted a quantitative cross-platform topic modeling of discussions about immigrants in online Singaporean communities (Study 1) to understand the expressive use of social media to discuss immigrants in Singapore. We supplemented the analyses on the expressive use of social media with a survey-based examination (Study 2) of the threats associated with the informational use of social media for anti-immigrant affect and the moderating variables that affect these relationships.

Our findings offer two main theoretical contributions. First, they address a preeminent research gap suggesting the theoretical link between cognitive ability and prejudice (Brandt and Crawford, 2016). Furthermore, while ITT traditionally focuses on cognitive prejudice, our findings emphasize the importance of affective prejudice, such as feelings of contempt, disgust, anger, envy, and jealousy. This broadens the understanding of prejudice from mere cognitive evaluations to more visceral emotional reactions, suggesting that social media shapes people’s thoughts about outgroups and how they emotionally respond to them.

A second contribution is the empirical evidence for the relationship between social media use and negative emotions toward outgroups. By their algorithms and user behaviors, social media platforms often present a skewed or biased view of outgroups, leading to heightened threat perceptions (Guess et al., 2023). Our findings corroborate the implications from Guess et al. (2023) to suggest that it is not just the algorithms encountered nor the sheer volume of social media use that matters, but who uses them and how they perceive the threats in the content and discussion they encounter.

Our research recommends using multi-method approaches to offer a comprehensive understanding of different media, such as social media, in terms of their role as a space for building and influencing public opinion. Findings related to heightened affective prejudice in Singapore emphasize the need to foster cross-group harmony through interactions and communities on social media and possibly other public spaces that eschew the social and economic constraints evident in offline societies. We recommend that policy efforts focus on literacy as a supplementary way to improve ingroup-outgroup relationships. Dispelling prevailing stereotypes through fact checks and educational efforts on social media may offer a more viable alternative than efforts to curtail social media content altogether.

## Data availability statement

The raw data supporting the conclusions of this article will be made available by the authors, without undue reservation.

## Ethics statement

The studies involving humans were approved by the Institutional Review Board, Nanyang Technological University. The studies were conducted in accordance with the local legislation and institutional requirements. Written informed consent for participation in this study was provided by the participants. Social media analyses were conducted on anonymized posts, aggregated from all the sources, to minimize the risks of accidental deidentification.

## Author contributions

SA: Conceptualization, Data curation, Formal analysis, Investigation, Methodology, Writing – original draft, Writing – review & editing, Project administration. KJ: Conceptualization, Data curation, Formal analysis, Methodology, Writing – original draft, Writing – review & editing, Investigation. VC: Data curation, Project administration, Writing – review & editing. MC: Writing – original draft. AChe: Writing – original draft. CE: Data curation, Methodology, Writing – original draft. VY: Writing – original draft. AChi: Funding acquisition, Project administration, Writing – review & editing.
